# Targeting Oxidative Stress in Intracerebral Hemorrhage: Prospects of the Natural Products Approach

**DOI:** 10.3390/antiox11091811

**Published:** 2022-09-14

**Authors:** Yingyi Zheng, Ruoqi Li, Xiang Fan

**Affiliations:** School of Basic Medical Sciences, Zhejiang Chinese Medical University, Hangzhou 310053, China

**Keywords:** oxidative stress, intracerebral hemorrhage, natural products, antioxidants, stroke

## Abstract

Intracerebral hemorrhage (ICH), the second most common subtype of stroke, remains a significant cause of morbidity and mortality worldwide. The pathological mechanism of ICH is very complex, and it has been demonstrated that oxidative stress (OS) plays an important role in the pathogenesis of ICH. Previous studies have shown that OS is a therapeutic target after ICH, and antioxidants have also achieved some benefits in the treatment of ICH. This review aimed to explore the promise of natural products therapy to target OS in ICH. We searched PubMed using the keywords “oxidative stress in intracerebral hemorrhage” and “natural products in intracerebral hemorrhage”. Numerous animal and cell studies on ICH have demonstrated the potent antioxidant properties of natural products, including polyphenols and phenolic compounds, terpenoids, alkaloids, etc. In summary, natural products such as antioxidants offer the possibility of treatment of OS after ICH. However, researchers still have a long way to go to apply these natural products for the treatment of ICH more widely in the clinic.

## 1. Introduction

Intracerebral hemorrhage (ICH), the second most common subtype of stroke, remains a significant cause of morbidity and mortality worldwide, although our understanding of its underlying pathologic mechanisms has progressed rapidly in the last two decades [1]. Approximately 15% of all stroke cases are ICH, but the mortality and morbidity in ICH patients exceed that of ischemic stroke patients [2]. ICH occurs when the arterial vasculature ruptures for various reasons, causing blood to leak into adjacent tissues [3]. Presently, there is no pharmacological or surgical treatment that could significantly improve neurological function after ICH [4]. Large numbers of experimental studies demonstrated that the interaction of cytotoxicity, excitotoxicity, oxidative stress (OS), and inflammation generated by the products of red blood cell lysis and plasma components caused subsequent brain injury after ICH [5]. However, the precise pathophysiological mechanisms underlying ICH remain to be completely elucidated.

Helmut Sies came up with the term “oxidative stress” to describe the imbalance between the generation of oxidants and antioxidant defenses that could cause harm to biological systems [6]. OS is related to several disease processes and refers to an imbalance in the oxidative and antioxidant actions of the body. According to substantial evidence, OS is implicated in the pathophysiology of numerous brain diseases, including neurodegenerative disorders, depression, and ischemic stroke [7]. Importantly, OS is also a primary mediator of secondary brain injury following ICH [8]. Experimental results have indicated that OS plays a critical role in brain damage after ICH, even though clinical trial results have been disappointing [9]. Consequently, there are currently no clinically available drugs for protecting the brain from OS injury following ICH. In recent years, researchers have taken a keen interest in natural products as potential new stroke drug candidates because of their positive effects on brain injury prevention in animal stroke models. In this review, we focus on the sources of free radicals after ICH; we also summarize in detail the natural products of antioxidant therapy in the ICH model. For this review article, we searched PubMed using the keywords “oxidative stress in intracerebral hemorrhage” and “natural products in intracerebral hemorrhage.”

## 2. The Pathogenesis of ICH-Induced Brain Damage

One of the most common causes of hemorrhagic stroke is the rupture of blood vessels in the brain, which could lead to both intracerebral and subarachnoid hemorrhage (SAH) [10]. The injuries caused by ICH mainly include primary injury and secondary injury. Primary brain injury is defined as bleeding caused by non-traumatic intraparenchymal vascular rupture. Hematoma formation occurs after ICH in the acute phase and can increase intracranial pressure, which subsequently compresses the surrounding tissue, thereby affecting blood flow to form ischemia and leading to brain herniation [11]. The size of the hematoma following ICH is not static; it continues to increase, pulling on the surrounding nerve fibers and causing compression on the surrounding tissues, which can cause mechanical damage to the brain tissue, a condition known as mass effect [12]. Hematoma expansion leads to midline shift and further neurological deterioration [13]. Meanwhile, hematoma mechanical compression and toxic compounds created by blood clots trigger neuronal death, disrupt the blood-brain barrier (BBB), and result in cerebral edema [14].

**Figure 1:**
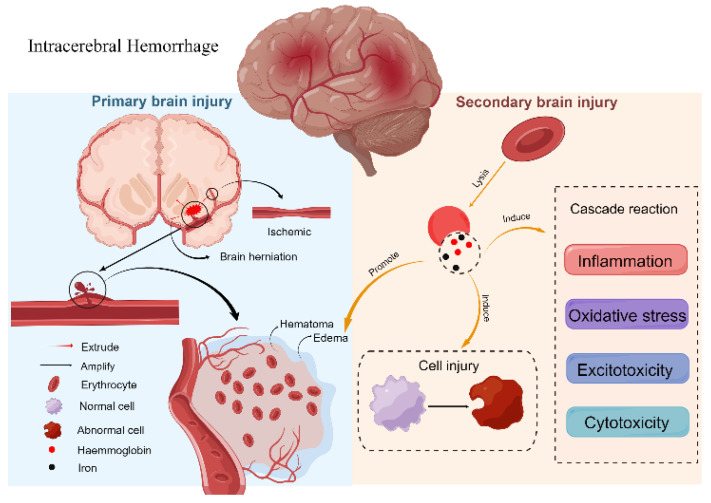
Mechanisms following intracerebral hemorrhage (figure made by Figdraw).

As shown in Figure 1, secondary injury following ICH could be induced by a chain reaction of events triggered by the primary injury, the physiological response to the hematoma, and the release of clot components. Inflammation, OS, excitotoxicity, and cytotoxicity are all components of secondary injury [11]. This cascade occurs minutes after a brain hemorrhage and lasts for days, weeks, or even months [15]. After ICH, thrombin secreted by the body will stop the hemorrhage, and the hematoma tends to be stable. Thrombin is a double-edged sword that can prevent bleeding and inflict nerve and endothelial cell damage [16]. As we all know, the main component of hematoma is red blood cells. Red blood cells release hemoglobin, iron, and heme, which are the primary causes of secondary injury and are linked to OS [17].

## 3. OS in ICH

**Figure 2:**
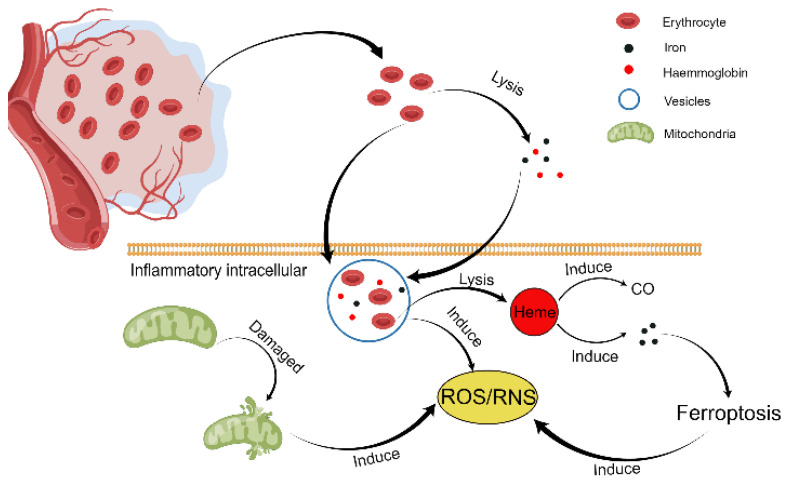
Source of ROS/RNS after intracerebral hemorrhage (figure made by Figdraw).

Humans require oxygen for survival, and when they consume oxygen for metabolism, they release certain charged molecules known as free radicals. Free radicals include reactive oxygen free radicals (ROS) and reactive nitrogen free radicals (RNS), of which reactive oxygen species are the primary source of free radicals. ROS includes superoxide anion radical (O_2_^•−^), hydroxyl radical (·OH) and hydrogen peroxide (H_2_O_2_). RNS includes nitric oxide (NO) and nitrogen dioxide (NO_2_). The damage caused by OS to the body is mainly ROS, which is mainly caused by two different effects: first, due to its unstable and highly reactive chemical properties, ROS will react with lipids, proteins, and DNA, resulting in body or cell senescence and death; second, in contrast, ROS is involved in cell homeostasis functions through the heat shock transcription factor 1 (HSF-1), nuclear factor-κB (NF-κB), phosphoinositide 3-kinase, and mitogen-activated protein [18]. OS is a situation in which the human body responds to damaging stimuli by producing excessive ROS and RNS. Free radicals have the characteristics of high activity and unstable chemical properties, so they will seize the electrons carried by other molecules to make them more stable. Free radicals could destroy cell walls, tunica intima, proteins, lipids, and DNA molecules, leading to cell or tissue damage. The brain is high in lipids but low in antioxidants such as superoxide dismutase (SOD), making it especially susceptible to OS damage [19]. Specifically, the brain is more susceptible to oxidative stress-induced damage for the following reasons: (1) the cellular oxygen consumption in the human brain accounts for 20% of the total oxygen consumption of the body, but the brain weighs only 2% of the body, indicating that the free radicals produced by the brain are significantly greater than those produced by other organs; (2) the brain has a lot of iron compared to other organs, and iron can catalyze the generation of free radicals; (3) in comparison to the kidney or liver, the brain is rich in lipids with unsaturated fatty acids, which are targets for lipid peroxidation, and the brain has low to moderate protective antioxidant systems [20]. Based on the above reasons, OS is crucial in secondary injury after ICH and is involved in all critical stages of the pathophysiological response after ICH (as shown in Figure 2).

Following ICH, the primary sources of ROS are the activated neutrophils, microglia, and macrophages. The most prominent source of these is microglia (Figure 2). ROS are incredibly active and have a short half-life, making laboratory analysis extremely difficult [21]. OS is usually evaluated indirectly by measuring the oxidized products of macromolecules, such as 8-Hydroxy-2-deoxyguanosine (8-OHdG), malondialdehyde (MDA), 4-hydroxy-trans-2-nonenal (4-HNE), dinitrophenyl (DNP), etc. The 8-OHdG is an oxidative adduct produced by reactive oxygen radicals (such as hydroxyl radicals and singlet oxygen species) attacking the carbon atom at position 8 of the guanine base in DNA molecules, which can cause G-C/A-T base pairing errors during DNA replication if not removed in time and thus cause gene point mutations or even carcinogenesis [22]. Therefore, it was used to evaluate the extent of DNA damage following OS. In organisms, free radicals act on lipids to undergo peroxidation, and the oxidative end products are MDA and acetaldehyde, which can cause cross-linking polymerization of life macromolecules such as proteins and nucleic acids, and are cytotoxic. The body produces oxygen free radicals through enzymatic and non-enzymatic systems, which attack polyunsaturated fatty acids in biofilms, trigger lipid peroxidation, and thus form lipid peroxidation acids. Lipid peroxidation not only converts ROS into reactive chemicals, i.e., lipid decomposition products with a non-free base, but also amplifies the action of ROS through chain or chain branch chain reactions. Therefore, an initial reactive oxygen species can lead to the formation of many lipid decomposition products, some of which are harmless, while others can cause cell metabolism and dysfunction, and even death. Oxygen free radicals can cause cell damage not only through the peroxidation of polyunsaturated fatty acids in biofilms but also through the decomposition products of lipid hydroperoxides [22]. The lipid oxidation final product MDA affects the activity of the mitochondrial respiratory chain complex and key enzymes in mitochondria in vitro, and its production can also aggravate membrane damage, so testing the amount of MDA can reflect the degree of lipid peroxidation in the body and indirectly reflect the degree of cell damage [22]. OS-induced lipid peroxidation produces not only MDA but also 4-HNE, which changes the fluidity and permeability of cell membranes and ultimately leads to changes in cell structure and function. Therefore, it is frequently used to judge indicators of lipid peroxidation. The level of DNP can represent the degree of protein damage after OS, so researchers often quantify it by Western blot [22]. In the ICH model of rats, 8-OHdG and DNP around the hematoma increased simultaneously and peaked on the third day [23]. These oxidative indicators have been found to be elevated in ICH animal models and human patients [24].

## 4. The Main Source of free Radicals following ICH

### 4.1. Mitochondria

Physiologically, a portion of the electrons in the electron transport chain of the inner mitochondrial membrane are detached, and roughly 1% to 2% of oxygen is oxidized to superoxide anions. Under situations of external stimulation, mitochondria sustain damage and create more ROS. In a normal situation, ROS could be eliminated by the body itself, which helps to maintain homeostasis. ROS are produced in large quantities as a result of mitochondrial malfunction, which happens during ICH. Research demonstrated that a mitochondrial ROS-specific scavenger could reduce ROS after ICH [25]. ROS generation in mitochondria could be attributed to the opening of inner membrane anion channels and mitochondrial permeability transition pore, resulting in changes in the intracellular and intramitochondrial oxidation environments, which trigger the release of ROS. It was discovered that blocking the activation of mitochondrial permeability transition pore and neutralizing the excessive generation of mitochondrial ROS eased OS damage caused by ICH [25]. A study on mitochondrial dysfunction after ICH showed that a mitochondrial ROS-specific scavenger could significantly alleviate the increased ROS following ICH [25].

### 4.2. Hemoglobin

Hemoglobin (Hb) is the major erythrocyte breakdown product and the principal mediator of oxidative damage following ICH [26]. Studies have demonstrated that a high level of ROS is generated after exposing Hb to cell culture in vitro or injecting Hb into mouse striatum in vivo [27]. There are different statements about Hb promoting oxidative damage. Some argued that iron released during Hb degradation causes oxidative damage because iron chelators could prevent Hb-induced neurotoxicity. Some claimed that when hemoglobin broke down naturally into oxyhemoglobin and methemoglobin without the aid of enzymes, Hb could release a great deal of superoxide [28].

### 4.3. Heme

Hb, a combination of globin and heme, is the most critical component of red blood cells. After ICH, heme is encapsulated in the hydrophobic pocket of Hb, which in turn is encapsulated in red blood cells. Hb degrades large amounts of heme, which could produce enormous amounts of ROS, so heme is cytotoxic [29]. When it comes to heme catabolism, heme oxygenase is both the starting point and the limiting factor [30]. Three isoenzymes of heme oxygenase (HO) exist: HO-1, HO-2, and HO-3 [31]. It has been well-established that three mechanisms contribute to the cytotoxicity of heme [31]. It initiates free radical chain reactions by decomposing preformed lipid peroxides, which oxidize membrane lipids rapidly and efficiently [32]. Second, it disrupts membrane stability through an as-yet-unidentified colloid osmotic mechanism that is unaffected by antioxidants [32]. Third, the breakdown of hemin by the heme oxygenase enzymes may cause iron-dependent oxidative damage to cell populations that cannot store large amounts of iron, such as neurons [33]. Studies have shown that the co-culture of astrocytes and endothelial cells with heme in vitro produced a significant increase in ROS, which was also observed in rats injected with heme intracerebroventricularly [34].

### 4.4. Iron

As one of the most significant hematoma degradation products, iron could cause direct toxic damage and DNA damage in the acute phase of ICH and cognitive impairment in the chronic phase of ICH [35]. The researchers found that, in a rat model of ICH with intraventricular collagenase injection, iron was detected around the hematoma on the first day after ICH, peaked on day 7, and remained at a higher level at 14 days [34]. In another study, the researchers observed a similar trend in a mouse ICH model using the intraventricular injection of autologous blood and collagenase; that is, iron around the hematoma began to increase at 3 days after ICH, peaked at 14 days, and decreased at 28 days [35]. Iron overload induces oxidative damage via the Fenton reaction, which generates ROS, particularly toxic hydroxyl radicals [36]. In rats injected intracerebroventricularly with FeCl_2_, oxidative damage to DNA was observed, demonstrating directly that iron mediates oxidative damage [37]. After ICH, it is indicative of iron-mediated oxidative damage that both ROS and 8-OHdG are significantly reduced when iron chelators are administered to ICH rats [38].

### 4.5. Inflammatory Cells

An important factor leading to secondary injury in ICH is the infiltration of inflammatory cells. After ICH, microglia are the first cells to respond and activate within minutes to release cytokines and chemokines and recruit neutrophils from the peripheral blood to aggravate brain injury [39]. In the inflammatory reaction, activation of neutrophils results in a respiratory chain burst and release of large amounts of ROS, which leads to high consumption of SOD produced by the body, OS imbalance, lipid peroxidation, and brain injury [40]. In rats with ischemic stroke, depletion of neutrophils could reduce free radical generation. In ICH models, it has also been shown that neutrophils could cause damage due to OS when they get inside the brain [41].

Typically, microglia are in a resting state (M0), and when ICH, it turns from a resting state to pro-inflammatory (M1) and anti-inflammatory (M2) activation phenotypes [42]. At the same time, the two phenotypes could be interconverted. Studies have shown that Hb could promote microglial activation through toll-like receptors [43]. ROS is also released when the phenotypes of M1 and M2 microglia are out of balance [44]. Microglia exposed to erythrocyte lysis produce more ROS in vitro [45]. Furthermore, in animal models of ICH, inhibiting microglial expression could reduce ROS and edema by a large amount and improve neurological function and neuronal activity [46]. Microglial activation is also associated with OS-related genes, such as nitric oxide synthase, COX2, tumor necrosis factor (TNF-α), and interleukin 1β [47].

## 5. Targeted Therapy of Oxidative Stress with Natural Products

Researchers are becoming more interested in traditional and alternative medicines, particularly natural products, as a result of the lack of effective and widely applicable pharmacological strategies for the treatment of ICH [48]. Numerous animal and cell studies on ICH have demonstrated the potent antioxidant properties of natural substances [49]. Despite the success of natural products in animal and cell experiments, there is still much work to be done before they can be used in clinical settings. The following table provides a list of studies of natural products that are effective antioxidants in ICH models (Table 1).

### 5.1. Polyphenols and Phenolic Compounds

Polyphenols refer to a collective term for chemical elements in a group of plants, each named for having multiple phenolic groups. Natural polyphenols are mainly found in fruits and vegetables, nuts, soybeans, tea, cocoa, and alcohol. Polyphenols are known as “the seventh category of nutrients” and have antioxidant effects [91]. As shown in Table 1, phenolic compounds could attenuate the oxidative stress caused by ICH. 

#### 5.1.1. Baicalein and Baicalin

Baicalein, the aglycon of baicalin, is a substance extracted from *Radix Scutellariae*, which has the functions of antioxidation, antitumor, and neuroprotection [92]. Baicalein protected rats with ICH and SAH, which could reduce vasospasm, edema, and the size of the hematoma and increase the number of neurons that survive [50,51]. In addition, many widely used oxidative stress indicators revealed that the antioxidant function of baicalein was crucial [50]. Baicalein also reduced MDA levels after SAH by preserving the activities of SOD and catalase (CAT). Baicalein increased SOD and glutathione peroxidase (GSH-Px) activity while decreasing MDA levels in the brain tissues of ICH model rats [51].

Similarly, baicalin is also an essential component in *Radix Scutellariae*. A pharmacokinetic study showed that baicalin could cross the BBB and improve neurological impairment in mice (specific data are presented in Table 1) [52,93]. Baicalin could reduce ROS and oxidative damage induced by ferroptosis, which has been confirmed through in vivo and in vitro experiments on ICH [94]. Together, these results demonstrated that baicalein and baicalin could be used to treat ICH injury by targeting oxidative stress.

#### 5.1.2. Curcumin

Curcumin is a hydrophobic polyphenolic compound derived from *Curcuma longa* with the properties of antioxidation, anticancer, and antiviral activities [95]. Numerous studies have shown that curcumin has significant potential in cardiovascular and cerebrovascular diseases [96]. Previous reports have demonstrated that curcumin has therapeutic effects on ischemic stroke in vitro and in vivo, although curcumin has poor water solubility, poor oral availability, and cannot penetrate the BBB [97]. Curcumin could reduce the production of superoxide, ROS, and MDA and increase the content of SOD and CAT in SAH animal models [53,54,55]. In the SAH mouse animal model, the lowest effective dose of curcumin was 150 mg/kg [53,54]. Curiously, the lowest effective dose in this literature was 20 mg/kg [55]. Some researchers have turned curcumin into nanoparticles that reduce ROS production and increase SOD, CAT, and GSH-Px levels in the SAH model [56,57,58]. In the SAH model in vitro, the antioxidant effect of curcumin was also confirmed, and curcumin could decrease the production of ROS and MDA and increase the content of SOD and GSH-Px [59].

#### 5.1.3. Luteolin

Luteolin is found everywhere in nature and can be extracted from medicinal plants and fruits. Luteolin received its name because of being extracted firstly from the leaves, stems, and branches of *Reseda odorata* L., which is in the Resedaceae. Modern pharmacological studies have shown that luteolin has anti-inflammatory, antioxidant, anti-apoptosis, antitumor, and autophagy-regulating effects [98,99,100,101]. Luteolin has antioxidant effects because it can decrease the production of ROS and MDA and increase the production of SOD, GSH-Px, CAT, HO-1, and glutathione (GSH) [60,61]. The Nrf2/Keap-1 signaling pathway is the primary pathway through which luteolin can reduce oxidative stress after SAH [60,61].

#### 5.1.4. Quercetin

Quercetin is a flavanol compound found in many plants that performs various biological functions. Quercetin is the most common flavonoid in nature [102]. Because of this, it is also the polyphenolic compound that has been studied the most. Quercetin has poor water solubility, unstable chemical properties, and a short biological half-life, which limit its clinical application [103]. Quercetin has been approved by the U.S. Food and Drug Administration (FDA; National Drug Code number is 65,448–3085–3005) because it fights free radicals and allergies, even though it has many of the problems listed above [104]. The results showed that quercetin could be detected in the brains of rats after oral administration at 100 mg/kg, indicating that quercetin could cross the BBB (specific data are presented in Table 1) [105]. In the SAH model, quercetin may alleviate brain damage and provide neuroprotection by increasing the activity of endogenous antioxidant enzymes and inhibiting free radical generation [62,106]. Quercetin could reduce ROS and MDA production in a rat model of SAH with autologous blood [106]. Another study demonstrated that quercetin could enhance the activities of GSH-Px and copper/zinc superoxide dismutase (CuZn-SOD) and significantly decrease the level of MDA [62]. The researchers overcame the low oral availability of quercetin by making quercetin into a quercetin-loaded nanoemulsion, which could reduce the hematoma while maintaining glutathione S-transferase (GST) activity, increasing GSH content and overall antioxidant capacity [63].

#### 5.1.5. (−)-Epicatechin

(−)-Epicatechin (EC) is a natural plant flavanol compound belonging to the subgroup of flavan-3-ols [107]. EC is widely found in daily edible fruits or beverages, such as apples, grapes, tea, and red wine [108]. Pharmacological studies have shown that EC has antioxidant, lipid-lowering, hypoglycemic, and cardiovascular disease prevention effects and can cross the BBB when given intravenously (specific data are presented in Table 1) [64]. In a rat model of collagenase-induced ICH, EC could decrease perihematomal HO-1 protein expression as well as ROS-induced DNA damage (hydroethidine), lipid peroxidation (MDA), and protein oxidation (dinitrophenyl hydrazone) [65]. EC exerted antioxidant effects by up-regulating Nrf2 and phase II enzymes (SOD1 and NADPH quinone oxidoreductase 1 (NQO1)) [65]. The researchers further validated the above effects through an ICH mouse model with Nrf2 knockout and found that EC protected astrocytes from hemoglobin toxicity by up-regulating Nrf2 and inhibiting AP-1 activity [109].

#### 5.1.6. Silymarin

Silymarin, a natural flavonoid lignan compound, is a natural active substance extracted from the dried fruits of the Asteraceae plant *Silybum marianum*, and its main components are silybin, isosilybin, silydianin, and silychristin [110]. Silymarin is called a “natural liver protection drug”, but it also has antioxidant, antitumor, anti-cardiovascular disease, and other effects [66]. Silymarin played an antioxidant role in a rat model of ICH by preventing SOD, CAT, GSH, and GST activities to achieve the purpose of the antioxidant [86]. Moreover, Silymarin also decreased ROS and MDA levels and up-regulated the expression of Nrf2 and HO-1 [66].

#### 5.1.7. Astragaloside IV

Astragaloside IV (AS-IV) is extracted from the herb *Radix Astragali.*, which is a standard for evaluating the quality of *Radix Astragali*. The study of stroke in AS-IV is mainly focused on ischemic stroke, while there are few studies on ICH [111]. However, AS-IV has shown strong antioxidant, anti-apoptotic, and immune-enhancing effects in other diseases [111]. However, there is one study on AS-IV for OS injury induced by SAH [67]. AS-IV could reverse the up-regulation of MDA and down-regulation of SOD and GSH-Px induced by SHA [67].

#### 5.1.8. Puerarin and Naringin

Both puerarin and naringin are flavonoids belonging to the same polyphenolic compound. Puerarin was extracted from the traditional Chinese medicine *Pueraria lobata* (Willd) in 1950, and since then its pharmacological effects have been extensively studied [112]. Intravenous injection of puerarin could penetrate the BBB and exert the potential neuroprotective effects in central nervous systems (CNS) disorders such as ischemic stroke, Alzheimer’s disease (AD), SAH, and Parkinson’s disease (PD) [68,113,114,115]. After intraperitoneal injection of puerarin in rats, puerarin could be detected in the hippocampus, cortex, and basal ganglia (specific data are presented in Table 1) [116]. In ICH animal models employing intracerebroventricular collagenase injection, Puerarin could significantly reduce the activity of 3-NT, 8-OHdG, and ROS, and this phenomenon has also been observed in SAH models [68,69].

In 1857, De Vry first found naringin in grapefruit blossoms [117]. Since then, naringin has been found in various fruits, vegetables, and nuts, such as grapes, cherries, tomatoes, beans, and cocoa [117]. Although its oral availability is poor, naringin could easily penetrate the BBB (specific data are presented in Table 1), and the anticancer, antibacterial, and antioxidant effects of naringin have been demonstrated [70,117,118]. Administration of naringin reversed ICH-induced decreases in enzymatic activity of SOD and CAT, levels of GSH, and increases in MAD and ROS levels [70].

#### 5.1.9. Gastrodin

Gastrodin, a phenolic glycoside, is an organic compound extracted from the dried roots of the orchid plant *Gastrodia elata Blume*. Studies have shown that gastrodin could penetrate the BBB (specific data are presented in Table 1), which provided a theoretical basis for the potential protective effects on CNS diseases [119,120]. Gastrodin has the effect of dilating blood vessels, and it is the only drug for the treatment of vertebrobasilar insufficiency in clinical practice. With the continuous deepening of research, the antioxidant, anti-apoptotic, and sedative effects of gastrodin have also been confirmed [121].

In recent studies, gastrodin could significantly reduce ROS levels caused by ICH and reduce ICH-induced increase of oxidative damage marker of lipid (MDA), protein (3-NT), and nucleic acid (8-OHdG) at 72 h following ICH [71]. Moreover, gastrodin significantly increased the expression of keap-1, Nrf2, and HO-1 and increased the activities of SOD and GSH-Px enzymes, demonstrating that gastrodin reduces oxidative stress injury after ICH through the Nrf2/HO-1 pathway [71]. This is consistent with the previous antioxidant effect of gastrodin through the Nrf2/HO-1 pathway to reduce oxidative stress damage in other diseases [122,123].

### 5.2. Terpenoids

Terpenoids (isoprenoids) are compounds and their derivatives are derived from meprenoic acid, with the isoprene unit (five-carbon units) as the basic structural unit of the molecular skeleton. These oxygenated derivatives include alcohols, aldehydes, ketones, carboxylic acids, esters, etc. Terpenoids are widespread in nature and are the main components of fragrances, resins, and pigments that constitute certain plants. For example, rose oil, eucalyptus oil, turpentine, and other terpenoids are contained. In addition, hormones and vitamins in some animals also belong to terpenoids [124].

#### 5.2.1. Astaxanthin

Astaxanthin, a keto-carotenoid, is a terpene unsaturated compound with multiple uses, including dietary supplements and food dyes. Astaxanthin has a high antioxidant capacity, which allows it to scavenge singlet oxygen and free radicals, preventing lipid peroxidation [125]. Astaxanthin has a wide range of biological activities and effects due to its antioxidant properties and cell signal modulating properties. Astaxanthin is a kind of chain-breaking antioxidant with a strong antioxidant capacity, which could scavenge nitrogen dioxide, sulfide, disulfide, etc.; reduce lipid peroxidation; and effectively inhibit lipid peroxidation caused by free radicals [126]. The strong antioxidant activity of astaxanthin is because it could stabilize the structure of the membrane, reduce membrane permeability, and limit the entry of peroxide promoters into cells. Astaxanthin could protect important intracellular molecules from oxidative damage. Conjugated double bonds, hydroxyl groups, and unsaturated ketone groups at the ends of conjugated double bond chains in astaxanthin molecules have relatively active electron effects and could provide electrons to free radicals or attract unpaired electrons from free radicals, effectively dampening singlet ROS with powerful oxidation properties as well as other free radicals in the environment [126]. Because astaxanthin is lipid-soluble, it could easily penetrate the BBB and exert antioxidant effects [127]. Astaxanthin could be detected in the rat brain after oral administration (specific data are presented in Table 1) [127]. Astaxanthin therapies significantly reduced the increased MDA levels and restored the suppressed SOD and GSH levels at 30 min and 3 h after SAH [72]. The protection after astaxanthin treatment of SAH was mainly through the Nrf2-ARE pathway. Specifically, astaxanthin exerted antioxidant effects by up-regulating NQO1, Nrf2, and HO-1 [73]. The antioxidant properties of astaxanthin have all been demonstrated in both in vitro and in vivo models of ICH [128].

#### 5.2.2. Artemisinin

Artemisinin, a class of compounds extracted from *Artemisia annua* L., is a well-established drug for the treatment of malaria [129]. In addition to its role in the treatment of malaria, artemisinin has antibacterial, antioxidant, and protective properties, and it has also been demonstrated to protect the central nervous system [130]. Studies have shown that artemisinin could increase the expression of neural cell adhesion molecule L1 to help ICH mice recover from neurological damage, which could also lower the levels of ROS, 3-NT, 4-HNE, and 8-OHdG and boost the activities of GSH and SOD [74].

#### 5.2.3. Oleuropein

Oleuropein is a class secoiridoid compound isolated from the leaves of olive trees. Oleuropein has potent antibacterial and antiviral properties, as well as an extremely strong antioxidant capacity [131]. Oleuropein exhibited protective effects against a variety of diseases, such as ischemic stroke, AD, and nonalcoholic fatty liver disease [75]. The therapeutic effects of oleuropein on ICH rats were increased in a dose-dependent manner, which could significantly reduce the levels of MDA and ROS and increase the activities of SOD and GSH-Px [75].

#### 5.2.4. Parthenolide

Parthenolide is the primary extract of *Tanacetum parthenium*, which is the main component of sesquiterpene lactone. In the past, parthenolide was mainly used to treat migraines, fevers, and rheumatoid arthritis [132]. However, recent studies have shown that parthenolide could also play an antioxidant role [76,133]. The bioavailability of parthenolide is high, and only 0.5 mg/kg could reverse the increase in ROS and the decrease in SOD and GSH activities induced by ICH.

#### 5.2.5. Ursolic Acid

Ursolic acid is a triterpenoid found in natural plants, which has various biological effects such as sedation, antibiotics, antidiabetes, antiulcer, and lowering blood glucose, and ursolic acid also has a significant antioxidant function, so it is widely used as a raw material for medicine and cosmetics [134]. At the same time, ursolic acid is an antioxidant. It has been shown that ursolic acid could inhibit the activities of 5-lipoxygenase and cyclooxygenase in the process of arachidonic acid metabolism and prevent the production of prostaglandins and leukotrienes, which may be the reason why ursolic acid inhibited the inflammatory response and lipid peroxides [77]. In an experiment on the treatment of SAH with ursolic acid, MDA was significantly higher. At the same time, the activities of GSH, CAT, and SOD were decreased in the cerebral cortex of rats in the SAH group compared with the vehicle group. In contrast, the ursolic acid group adjusted the above parameters to normal levels [77].

#### 5.2.6. Bakuchiol

Bakuchiol is a prenylated phenolic monoterpene isolated from the seeds of *Psoralea corylifolia* L [135]. Bakuchiol was first extracted in 1997, and its pharmacological effects have been widely studied with antioxidant, antibacterial, antiageing, and anti-inflammatory effects [135]. A recent study showed that bakuchiol could reverse the increase of MDA, ROS, 3-NT, 8-OHdG, and 4-HNE induced by SAH. Meanwhile, bakuchiol could also increase the activities of SOD and GSH-Px, and the specific mechanism is to improve mitochondrial morphology through the Trx/NIP system to exert antioxidant function [78].

### 5.3. Alkaloids

Alkaloids are a group of essential organic compounds that contain nitrogen and are widely found in nature (mostly in plants but partially in animals). Most alkaloids have complex ring structures, and nitrogen is mostly contained in the ring and has significant biological activity, which is one of the essential active ingredients in Chinese herbal medicines [136].

#### 5.3.1. Dauricine

Dauricine is an isoquinoline alkaloid isolated from the Chinese herbal medicine *Rhizoma menispermi.* Modern pharmacological studies have shown that dauricine has neuroprotective effects in AD and ischemic stroke [137,138,139]. Compared with the ICH group, dauricine at 5 mg/kg, 10 mg/kg, and 15 mg/kg could reduce MDA and ROS and alleviate ICH-induced injury [79].

#### 5.3.2. Tetramethylpyrazine

Tetramethylpyrazine is an alkaloid monomer extracted from *Ligusticum chuanxiong* Hort, a traditional Chinese medicine, and is the main active ingredient of *Ligusticum chuanxiong* Hort. Tetramethylpyrazine has been shown to have several pharmacological properties over the last few decades and has been used to treat a wide range of diseases with excellent effects. Two-[[(1,1-dimethylethyl) oxidoimino]-methyl]-3,5,6-trimethylpyrazine (TBN), nitrone derivative of tetramethylpyrazine, has an extremely strong antioxidant effect, which could scavenge free radicals such as O_2_^−^, ·OH, and ONOO^−^ in vitro [140]. Tetramethylpyrazine also has antioxidant effects in SAH rats, which could reduce the production of 8-OHdG, 3-NT, and ROS by up-regulating the Nrf2/HO-1 pathway [80].

#### 5.3.3. Isorhynchophylline

Isorhynchophylline is an alkaloid compound isolated from *Uncaria rhynchophylla* that could lower blood pressure, relax blood vessels, and protect nerves from damage caused by ischemia. In ICH rats, isorhyncholine could attenuate ferroptosis induced by iron overload, increase the expression of glutathione peroxidase-4 (GPX-4), and decrease ROS, 4-HNE, and MDA production [81].

### 5.4. Others

#### 5.4.1. Allicin

Allicin is an organosulfur compound extracted from the bulbs of allium sativum, a member of the alliaceae family, and is also found in onions and other alliaceae. The effects of allicin on cardiovascular disease and neuroinflammatory and degenerative diseases have been widely reported [141]. Allicin has also been reported to exert protective effects on the brain [82,142]. Meanwhile, allicin also showed antioxidant effects in SAH rats and could decrease the level of MDA and increase the activities of SOD and GSH [82].

#### 5.4.2. Cordycepin

Cordycepin is the first nucleoside antibiotic isolated from *Cordyceps* [143]. Cunningham et al., German scientists, discovered the core component of Cordyceps militaris, “cordycepin”, in 1951, which was discovered to have antibacterial, antiviral, antitumor, and immunomodulatory properties [144]. Until now, researchers have explored the biosynthetic mechanism of cordycepin in cordyceps militaris and found for the first time that cordyceps militaris could synthesize the anticancer drug, pentostatin, which is used to protect the structural stability of the synthesized cordycepin [145]. In an experiment on cordycepin treatment of mice with ICH, cordycepin effectively reduced the level of MDA in brain tissue within 3 days after ICH and increased the levels of SOD, GSH, and CAT [83].

#### 5.4.3. Crocin

Crocin is a water-soluble carotene isolated from *Crocus sativus* L. Modern studies have shown that crocin has a good effect on a variety of central nervous system and cardiovascular system diseases, but also has anticancer, antioxidative, hepatoprotective, cholagogue, and antidiabetic effects, in addition to long-term use as spices, dyes, and food additives [146]. In a recent study, the content of MDA was significantly reduced following treatment with crocin, while the activities of SOD and GSH-px were clearly increased in the crocin-treated group compared to the ICH group [84]. Crocin also elevated the expression of Nrf2 and GXP-4 and alleviated ICH-induced lipid oxidation [84].

#### 5.4.4. Polydatin

Common foods such as grapes and red wine contain polydatin, a naturally occurring active component isolated from the traditional Chinese herb *Polygonum cuspidatum* [147]. Polydatin is a glycoside form of resveratrol with the following structural formula: 3,4,5-trihydroxystilbene-3-β-mono-D-glucoside, including two isomers: cis- Polydatin and trans- Polydatin [147]. Therefore, polydatin also has an extremely strong antioxidant function. Compared to the autologous blood-induced ICH model group rats, the polydatin group rats had less NO and MDA in brain tissue while having more SOD and GSH [85]. Furthermore, the relative expressions of Nrf2, NQO1, and HO-1mRNA were higher in the brain tissue of rats treated with polydatin than in the ICH groups [85].

#### 5.4.5. Green Tea and Red Tea

There is a record of tea consumption in Chinese history, and tea has become popular as a beverage worldwide. Tea is rich in catechol, catechin, vitamin E, flavonoids, and other substances; regular tea is beneficial to health. In an interesting study, both black and green tea were able to inhibit ICH-induced ROS production and boost GSH activity [86]. Additionally, giving ICH rats green tea in the short-term could reduce the amount of ROS in the hippocampus and improve the memories of rats [87].

#### 5.4.6. Chrysophanol

Chrysophanol, a natural anthraquinone, was used in the food and pharmaceutical fields. Chrysophanol is found in many traditional Chinese herbal medicines, such as *Radix et Rhizoma Rhei*, *Cassia obtusifolia* L., and *Polygonum multiflorum*, with great medicinal value [148]. In the autologous blood ICH model, chrysophanol could decrease MDA expression and increase SOD, GSH, and CAT expression [88].

#### 5.4.7. Phillyrin

Phillyrin is an extract of the dried fruit of *Forsythia suspensa*, a member of the Oleaceae family [149]. Modern pharmacology is not deep enough to study phillyrin, although some studies have shown that phillyrin has the effects of antioxidation and anticancer [149]. Phillyrin increased the expression of Nrf2, HO-1, NQO1, and SOD-1, and decreased the expression of MDA and ROS in the in vitro and in vivo ICH model [89].

#### 5.4.8. Momordica Charantia Polysaccharide

*Momordica charantia* has a long history as a vegetable and is widely used in Asian herbal medicine, which contains rich momordica charantia polysaccharide, and has been shown to have pharmacodynamic functions such as anticancer, antioxidation, and improving immunity [150]. It has been demonstrated that momordica charantia polysaccharide could scavenge free radicals, decrease ICH-induced ROS and MDA expression, and increase SOD levels to exert neuroprotective effects after ICH [90].

## 6. Discussion

Although natural products perform well as antioxidants in ICH animal experiments, there are few clinical applications. Modern medical animal experiments are usually the default gold standard for preclinical evaluation, but the results of animal experiments are very poor or even opposite to the results of human clinical application, mainly for the following three reasons: (1) the effect of the laboratory environment and other changes on the study results; (2) the differences between animal models of diseases and human diseases; (3) the physiology and genetic differences between species [151]. At the same time, the pathological mechanisms of ICH are very complex, and there are still some limitations in the current study of the mechanisms, which also limit the development of clinical drugs. Although the efficacy and targets of natural products are relatively clear, the research on druggability is still insufficient. In addition, the animals used in previous studies were young and healthy, which may not mimic the actual clinical practice situation because ICH frequently occurs in the elderly, and patients may also suffer from other diseases. Due to the complex pathological mechanisms, the treatment of ICH should be multi-targeted agents. For example, a recent study showed that the combination of resveratrol and quercetin alleviated the production of pro-inflammatory factors, which could be one of the research directions for ICH in the future [152]. We should integrate network pharmacology, metabolomics, and genomics to understand better the advantages of the multi-target combination of natural products in the treatment of diseases in the future. Finally, we observed a phenomenon in which some natural products did not have data to cross the BBB, but they did play a role in the treatment of ICH. We propose a hypothesis that some natural products may exert their effects in the treatment of ICH through the brain-gut axis. The gut and the brain are closely linked through the vascular system and the vagus nerve to connect the brainstem to part of the gut to form direct neural connections. Increasing evidence suggests that bacteria and microbiomes that survive in the gut do influence the production of PD, and PD causes specific gut microbial changes [153]. Therefore, we speculate that these natural products that do not penetrate the BBB may play an indirect role in the treatment of ICH by affecting the gut microbiota or the brain–gut axis. At the same time, we think this is also a direction for future research.

At the same time, plant drugs are more prone to hormesis effects. In the 1980s, hormesis was cited only 10 to 15 times a year in the Web of Science database, but more than 3000 times in 2020, indicating that hormesis research has been gradually gaining appreciation. In short, hormesis is the phenomenon in which chemicals have negative effects on organisms at high doses (for example, inhibition of growth and development) but positive effects (for example, stimulation of growth and development) at low doses [154]. When luteolin was administered to ICH animal models at a dose of 10 mg/kg, it increased keap-1 expression. However, when the dose was increased to 20 mg/kg, keap-1 expression was not increased compared with 10 mg/kg [61]. The same thing happened when puerarin was used to treat ICH, and increasing the dose of puerarin did not have a linear effect on ROS expression in ICH models at 100 mg/kg compared to 50 mg/kg [69]. Although this phenomenon has been observed during experiments exploring drugs for the treatment of ICH, no one seems to study hormesis systematically. We believe that this is also worth doing in the future. Hormesis provides new opportunities for improving clinical treatment options and raises dangerous problems that must be solved.

## 7. Conclusions

In summary, natural products such as antioxidants offer the possibility for the treatment of OS after ICH. Currently, there is no specific therapeutic agent for the treatment of ICH, while there are a lot of natural products in vegetables, fruits, and plants, which are a huge treasure trove for researchers who want to develop new drugs for ICH. However, researchers still have a long way to go to apply these natural products for the treatment of ICH more widely in the clinic.

## Figures and Tables

**Table 1:** Antioxidant activity of nature product in ICH related studies.

S. N.	Phytochemicals	Cell/Animal Model	Dosages and Methods of Administration in Animal Models	Antioxidation-Related Indexes	BBB Penetration Capability (Administration Mode and C_max_)	Ref
1	Baicalein	SAH ICH	30 mg/kg i.p. 10, 50 mg/kg i.p.	Up: SOD, CAT Down: MDA Up: SOD, GSH-Px Down: MDA		[50] [51]
2	Baicalin	ICH PC12	20 mg/kg, p.o. 5, 10,20 μM	Up: GPX-4 Down: ROS	yes, 100 mg/kg i.v. Brain 501.33 ± 115.94 μg∙mL^−1^/ng·g^−1^	[52]
3	Curcumin	SAH SAH (SD) SAH (Wistar) ICH (C57) ICH (Wistar) SAH (SD) Cortical Neurons	150, 300 mg/kg i.p. 150 mg/kg i.p. 20 mg/kg i.p. 5 mg/kg, p.o. 30 mg/kg, p.o. 150, 300 mg/kg i.p. 5, 10 μM	Up: SOD Down: MDA UP: SOD Down: MDA UP: SOD, CAT Down: MDA Down: ROS Up: CAT Down: MDA Up: SOD, GSH-Px CAT Down: ROS, MDA 8-OHdG Up: SOD, GSH-Px Down: MDA, ROS		[53] [54] [55] [56] [57] [58] [59]
4	Luteolin	SAH (SD) Cortical Neurons and microglia ICH (SD) Cortical neurons	10, 30, 60 mg/kg i.p. 5, 10, 25 mM 5, 10, 20 mg/kg 10 μM	Up: SOD, GSH, GSH-Px, Nrf2, HO-1 Down: MDA, ROS Up: Nrf2, NQO1, HO-1 Keap-1		[60] [61]
5	Quercetin	SAH (Wistar) SAH (SD)	10, 50 mg/kg i.p. 10, 50 mg/kg i.p.	Down: MDA Up: GSH-Px CuZn-SOD Down: MDA	yes, 100 mg/kg p.o. Brain 842.1 ± 508.4 mg/L	[62] [63]
6	(−)-Epicatechin	ICH (C57) microglia	5, 15, 45 mg/kg p.o. 1, 10, 100 μM	Up: Nrf2, NQO1 SOD1, HO-1 Down: MDA Up: Nrf2, HO-1 SOD1 Down: ROS	yes, 10 mg/kg i.v. Brain 8.92 ± 2.68 µg/mL	[64] [65]
7	Silymarin	ICH (C57)	200 mg/kg i.p.	Up: Nrf2, HO-1 SOD, CAR, GSH Down: ROS, MAD		[66]
8	Astragaloside IV	SAH (SD)	10 mg/kg i.p.	Up: SOD, GSH-Px Down: MDA		[67]
9	Puerarin	SAH (C57) ICH (SD)	100 mg/kg i.p. 50, 100 mg/kg i.p.	Up: SOD2 Down: ROS Down: 3-NT, ROS 8-OHdG	yes, i.p. Hippocampus (µg/mL) 80 mg/kg 3.35 *±* 0.55 40 mg/kg 2.09 *±* 0.31 20 mg/kg 1.58 *±* 0.24 Cerebral cortex (µg/mL) 80 mg/kg 4.48 *±* 0.86 40 mg/kg 3.56 *±* 0.61 20 mg/kg 1.73 *±* 0.24 Striatum (µg/mL) 80 mg/kg 1.93 *±* 0.37 40 mg/kg 1.55 *±* 0.17 20 mg/kg 1.03 *±* 0.22	[68] [69]
10	Naringin	ICH (Wistar)	10, 20, 40 mg/kg p.o.	Up: SOD, GSH, CAT Down: ROS, MDA	yes, 120 mg/kg femoral vein 0.64 ± 0.18μg/ml	[70]
11	Gastrodin	ICH (SD) Cortical Neuron	100 mg/kg i.p. 100 μM	Up: SOD, Nrf2, HO-1 Keap-1, GSH-Px Down: 8-OHdG, MDA 3-NT, ROS	yes, 200 mg/kg femoral vein Frontal cortex (µg/mL) 21.6 ± 6.0 Hippocampus (µg/mL) 24.3 ± 9.4 Thalamus (µg/mL) 22.0 ± 6.9 Cerebellum (µg/mL) 35.8 ± 10.3	[71]
12	Astaxanthin	SAH (SD) SAH (SD)	25, 75 mg/kg p.o. 20 μL i.p.	Up: SOD, GSH Down: MDA Up: Nrf2, HO-1 NQO-1 Down: MDA	yes, 100 mg/kg p.o. Hippocampus (pmol/g) 10.5 ± 1.1 Cerebral cortex (pmol/g) 19.6 ± 1.8	[72] [73]
13	Artemisinin	ICH (C57)	5 mg/kg i.p.	Up: GSH, SOD Down: 4-HNE, 3-NT 8-OHdG, ROS		[74]
14	Oleuropein	ICH (SD)	20, 40, 60, 80 mg/kg i.p.	Up: SOD, GSH-Px Down: ROS, MDA		[75]
15	Parthenolide	ICH (SD)	0.5, 1 mg/kg i.p.	Up: SOD, GSH Down: ROS		[76]
16	Ursolic acid	SAH (SD)	25, 50 mg/kg i.p.	Up: GSH, SOD, CAT Down: MDA		[77]
17	Bakuchiol	SAH (C57)	50 mg/kg orally by gavage	Up: SOD, GSH-Px Down: MDA, 3-NT 8-OHdG, 4-HNE		[78]
18	Dauricine	ICH (C57)	5 mg/kg i.p.	Down: ROS		[79]
19	Tetramethylpyrazine	SAH (SD) SAH (Rabbit)	60 mg/kg i.v. 30 mg/kg i.v.	Up: Nrf2, HO-1 Down: ROS, 3-NT 8-OHdG		[80]
20	Isorhynchophylline	ICH (SD) HT-22 cells	30 mg/Kg i.p. 30 μM	Up: SOD, GPX-4 Down: 4-HNE, MDA ROS		[81]
21	Allicin	SAH (SD)	30, 70 mg/kg i.p.	Up: SOD, GSH Down: MDA		[82]
22	Cordycepin	ICH (C57)	5, 10, 20 mg/kg i.p.	Up: SOD, CAT, GSH Down: MDA		[83]
23	Crocin	ICH (C57)	40 mg/kg i.p.	Up: SDO, GSH-Px GXP-4, Nrf2 Down: MDA		[84]
24	Polydatin	ICH (Wistar)	50 mg/kg i.p.	Up: Nrf2, NQO1, HO-1 SOD, GSH Down: MDA		[85]
25	Green or red tea	ICH (Wistar)	orally	Up: GSH Down: ROS		[86] [87]
26	Chrysophanol	ICH (Wistar)	10, 20 mg/kg, p.o.	Up: GPX, CAT, SOD GSH Down: MDA		[88]
27	Phillyrin	ICH (C57)	5, 15, 30 mg/kg i.p.	Up: Nrf2, NQO-1, HO-1 SOD-1, GSH Down: MDA, ROS		[89]
28	Momordica charantia polysaccharide	ICH (SD)	60, 75, 100 mg/kg i.p.	Up: SOD Down: MDA, ROS		[90]

## Abbreviations

ICH: intracerebral hemorrhage; OS, oxidative stress; SAH, subarachnoid hemorrhage; BBB, blood–brain barrier; ROS, reactive oxygen free radicals; RNS, reactive nitrogen free radicals; HSF-1, heat shock transcription factor 1; NF-κB, nuclear factor-κB; 8-OHdG, 8-Hydroxy-2-deoxyguanosine; MDA, malondialdehyde; 4-HNE, 4-hydroxy-trans-2-nonenal; DNP, dinitrophenyl; Hb, hemoglobin; HO-1, heme oxygenase-1; HO-2, heme oxygenase-2; HO-3, heme oxygenase-3; SOD, superoxide dismutase; TNF-α, tumor necrosis factor; GSH-Px, glutathione peroxidase; CAT, catalase; GSH, glutathione; CuZn-SOD, copper/zinc superoxide dismutase; GST, glutathione S-transferase; CNS, central nervous system; PD, Parkinson’s disease; AS-IV, astragaloside IV; EC, (-)-Epicatechin; NQO1, NADPH quinone oxidoreductase 1; GPX-4, glutathione peroxidase 4; Nrf2, nuclear factor erythroid 2-related factor 2.
